# Identification of the Important Genes of *Bradyrhizobium diazoefficiens* 113-2 Involved in Soybean Nodule Development and Senescence

**DOI:** 10.3389/fmicb.2021.754837

**Published:** 2021-11-11

**Authors:** Songli Yuan, Shunxin Zhou, Yong Feng, Chanjuan Zhang, Yi Huang, Zhihui Shan, Shuilian Chen, Wei Guo, Hongli Yang, Zhonglu Yang, Dezhen Qiu, Haifeng Chen, Xinan Zhou

**Affiliations:** ^1^Key Laboratory of Biology and Genetic Improvement of Oil Crops, Ministry of Agriculture and Rural Affairs of PRC, Oil Crops Research Institute of Chinese Academy of Agriculture Sciences, Wuhan, China; ^2^Shenzhen Branch, Guangdong Laboratory of Lingnan Modern Agriculture, Genome Analysis Laboratory of the Ministry of Agriculture and Rural Affairs, Agricultural Genomics Institute at Shenzhen, Chinese Academy of Agricultural Sciences, Shenzhen, China

**Keywords:** soybean symbiotic nitrogen fixation, soybean developmental stages, rhizobial gene expression, rhizobial DEGs, nodule development and senescence

## Abstract

Legume nodule development and senescence directly affect nitrogen fixation efficiency and involve a programmed series of molecular events. These molecular events are carried out synchronously by legumes and rhizobia. The characteristics and molecular mechanisms of nitrogen fixation at soybean important developmental stages play critical roles in soybean cultivation and fertilizer application. Although the gene expression of soybean were analyzed in nodules at five important soybean developmental stages, information on the expression of rhizobial genes in these nodule samples is limited. In the present study, we investigated the expression of *Bradyrhizobium diazoefficiens* 113-2 genes in the nodule samples from five developmental stages of soybean (Branching stage, flowering stage, fruiting stage, pod stage and harvest stage). Similar gene expression patterns of *B. diazoefficiens* 113-2 were existed during optimal symbiotic functioning, while different expression patterns were found among early nodule development, nitrogen fixation progress and nodule senescence. Besides, we identified 164 important different expression genes (DEGs) associated with nodule development and senescence. These DEGs included those encoding nod, nif, fix proteins and T3SS secretion system-related proteins, as well as proteins involved in nitrogen metabolism, ABC transporters and two-component system pathways. Gene Ontology, KEGG pathway and homology analysis of the identified DEGs revealed that most of these DEGs are uncharacterized genes associated with nodule development and senescence, and they are not core genes among the rhizobia genomes. Our results provide new clues for the understanding of the genetic determinants of soil rhizobia in nodule development and senescence, and supply theoretical basis for the creation of high efficiency soybean cultivation technology.

## Introduction

The symbiotic relationship between legume and soil rhizobia leads to the formation of root nodule and fixation of atmospheric nitrogen (Ferguson et al., 2010). Depending on the persistence of the nodule meristem and morphology characteristics of nodules, nodules in legumes can be divided into determinate nodules and indeterminate nodules (Hirsch, 1992). Nodules formed in *Glycine max* and *Lotus japonicus* are determinate nodules, they have non-persistent meristems and predominately contain one specific form of the bacterial development, which is determined by the nodule’s age (Karr and Emerich, 1988; Karr et al., 1990). Indeterminate nodules, such as those in *Medicago truncatula* and *Astragalus sinicus*, contain persistent meristems and a mixture of bacteroid forms in all stages of development (Vasse et al., 1990). In both determinate and indeterminate nodules, nodule development directly affects nitrogen fixation activity, and the premature senescence of nodules negatively regulates the efficiency of symbiotic nitrogen fixation (Serova et al., 2018). Delaying nodule senescence is a practical measure to increase nitrogen fixation amount and solve the problem of late defertilization in legume crops. Therefore, studies on the root nodule development and senescence are of great significance in the efficient use of nitrogen sources and legume crop yield.

Legume nodule development is initiated by an exchange of chemical signals between legumes and soil rhizobia, and included lots of protein-protein interactions between legumes and soil rhizobia proteins (Zhang et al., 2018). The leguminous molecular events involved in nodulation and nodule development are quite well understood (Li Q. G. et al., 2013; Mergaert et al., 2020; Roy et al., 2020). The characteristics and the corresponding detection methods, the key leguminous genes currently known to be involved in the regulation of nodule senescence and various abiotic factors affecting nodule senescence were summarized in our previous review (Zhou et al., 2021). Except for legumes, the soil rhizobia also play critical roles in symbiotic nitrogen fixation progress (Lindström and Mousavi, 2020). Because Nod factors (NFs), surface polysaccharides and secreted proteins are three main determinants of host specificity in most rhizobia, the rhizobial genes that affect the biological synthesis of these signaling molecules were explored in nodulation, including *nod*, *nif*, *fix* genes, surface polysaccharides biosynthesis genes and secretion system-related genes (Wang et al., 2014; Li et al., 2020). Besides, it has been demonstrated that several rhizobial genes play key roles in nodule senescence. *SmgshB* is a critical gene for the synthesis of glutathione, which is a carbon source that can ensure the normal development of nodules and can suppress the early senescence of nodules (Cheng et al., 2017; Yang et al., 2020). *lrpL-acdS* genes, which encode an ACC deaminase enzyme, are associated with nitrogen fixation and delayed nodule senescence (Tittabutr et al., 2015). The sigma factors *RpoH1* and *RpoH2* are involved in different stress responses and negatively regulate nodule senescence (Martínez-Salazar et al., 2009). *Anabaena variabilis* flavodoxin, a protein involved in the response to oxidative stress, can significantly delay nodule senescence in alfalfa (Redondo et al., 2009). Therefore, both leguminous genes and rhizobial genes are critical to nodule development and senescence.

Soybean (*Glycine max*) is an important leguminous crop and has been grown worldwide for edible oil, food and feed material (Palander et al., 2005). Large amounts of N from the atmosphere fixed by soybean symbiotic nitrogen fixation can be widely available for growth of soybean and other intercropping and rotation crops (Biswas and Gresshoff, 2014). Branching stage, flowering stage, fruiting stage, pod stage and harvest stage are five important developmental stages for soybean cultivation and fertilizer application studies, and the nitrogen fixation characteristics of these stages have been well studied (Fehr et al., 1971). In our previous study, the expression of nodule genes at above-mentioned five stages was assessed quantitatively using RNA-Seq, we only analyzed the important different expression genes (DEGs) of soybean in nodule development and senescence, but not of *Bradyrhizobium diazoefficiens* 113-2 (Yuan et al., 2017).

*B. diazoefficiens* 113-2 was collected from paddy fields in Hengyang area of Hunan Province, China in 1972 by Xuejiang Zhang, and its genome shared a large synteny blocks and a high ANI value with that of *B. diazoefficiens* USDA110 (Li et al., 2020). In this report, we investigated the expression of *B. diazoefficiens* 113-2 genes in the nodule samples from above-mentioned five developmental stages of soybean and identified 164 important DEGs associated with nodule development and senescence. These DEGs included those encoding nod, nif, fix proteins and T3SS secretion system-related proteins, as well as proteins involved in nitrogen metabolism, ABC transporters and two-component system pathways. Our results firstly connected the action mode of rhizobial genes during nodule development with the developmental stages of soybean, which should provide new clues for the understanding of the genetic determinants of soil rhizobia in nodule development and senescence, and shed new light on the molecular mechanisms of nitrogen fixation at above-mentioned five developmental stages of soybean.

## Results

### Expression Analysis of the Genes of *B. diazoefficiens* 113-2

**FIGURE 1:**
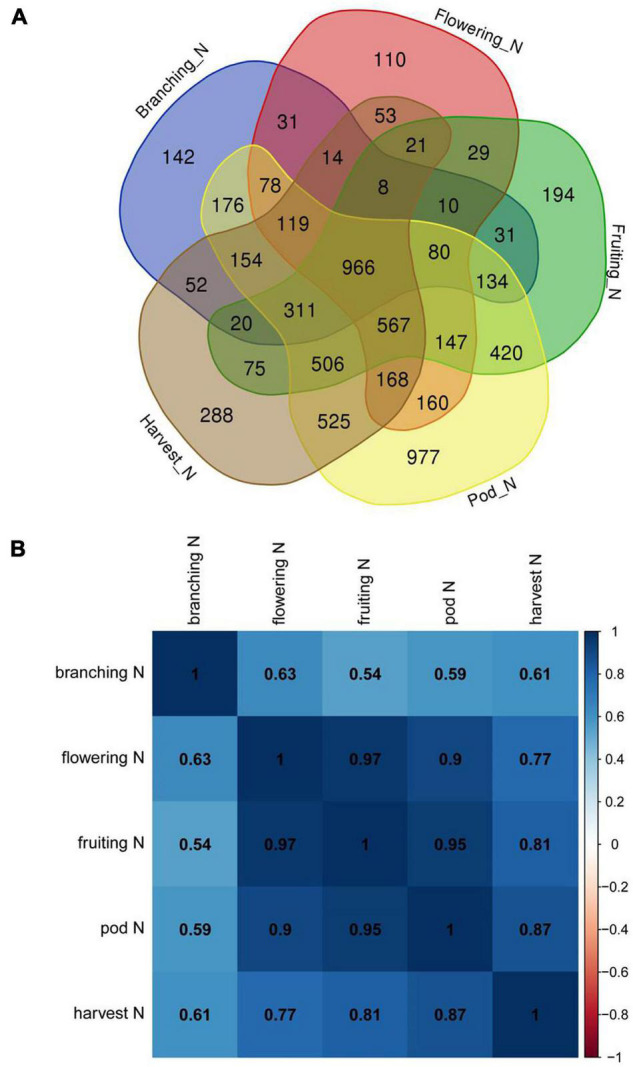
Expression pattern of genes of *B. diazoefficiens* 113-2 in soybean nodules at five developmental periods of soybean. **(A)** Venn diagram showing the number of genes identified in nodules at branching, flowering, fruiting, pod and harvest stages. **(B)** Correlation between different nodule samples according to the total gene expression levels.

We previously performed RNA-Seq analysis for five different nodule samples from five important development stages of soybean (Yuan et al., 2017) and sequenced the genome of *B. diazoefficiens* 113-2 (Li et al., 2020). In this report, we want to investigate the expression of the genes of *B. diazoefficiens* 113-2 in nodule development and senescence by using the above-mentioned RNA-Seq data. The genome and gene mapping details of *B. diazoefficiens* 113-2 were shown in Supplementary Table 1, and the results of sequencing saturation, reads coverage and reads random analyses were displayed in Supplementary Figures 1–3. As shown in Table 1, 2234 (about 25.38%) genes of *B. diazoefficiens* 113-2 were not identified in these five nodule samples, and more than 30% of the genes (except for Pod stage_N) contained meaningless fpkm values (0∼1). For the rest genes, most of the fpkm values were between 1 and 1,000, and a very small number of the fpkm values were above 10,000 (Table 1). Besides, the numbers of genes with meaningful fpkm values (>1) at one or more nodule samples were shown in Figure 1A. There are 966 genes consistently expressed at five nodule samples, 1085 genes were consistently found at four nodule samples, and 1252, 1552, and 1771 genes expressed at three, two and only one nodule sample, respectively. The detail fpkm information was shown in Supplementary Table 2.

**TABLE 1:** The numbers of *B. diazoefficiens* 113-2 genes in different fpkm value groups in five nodule samples.

	fpkm value				
	>10,000	1,000∼10,000	1∼1,000	0∼1	NA
Branching stage_N	16 (0.18%)	223 (2.53%)	2087 (23.71%)	4241 (48.19%)	2234 (25.38%)
Flowering stage_N	18 (0.20%)	181 (2.06%)	2362 (26.84%)	4006 (45.52%)	2234 (25.38%)
Fruiting stage_N	18 (0.20%)	208 (2.36%)	3293 (37.42%)	3048 (34.63%)	2234 (25.38%)
Pod stage_N	18 (0.20%)	210 (2.39%)	5260 (59.77%)	1079 (12.26%)	2234 (25.38%)
Harvest stage_N	25 (0.28%)	190 (2.16%)	3632 (41.27%)	2720 (30.91%)	2234 (25.38%)

To study the correlation of the expressions of *B. diazoefficiens* 113-2 genes among the five nodule samples, we calculated the Pearson correlation coefficients of each two nodule samples based on the total gene expression levels (Figure 1B). The Pearson correlation coefficients between Branching stage_N and the other four nodule samples were 0.54∼0.63, indicating that the expression of the genes of *B. diazoefficiens* 113-2 in Branching stage_N was very different from other four nodule samples. The Pearson correlation coefficients between harvest stage_N and flowering stage_N, fruiting stage_N or pod stage_N were less than 0.9, suggesting that relative lower correlation between these nodule samples. While the Pearson correlation coefficients among flowering stage_N, fruiting stage_N and pod stage_N were more than 0.9, indicating that relative higher correlation were existed in these three nodule samples.

### Different Expression Gene Identification Between Different Nodule Samples

**FIGURE 2:**
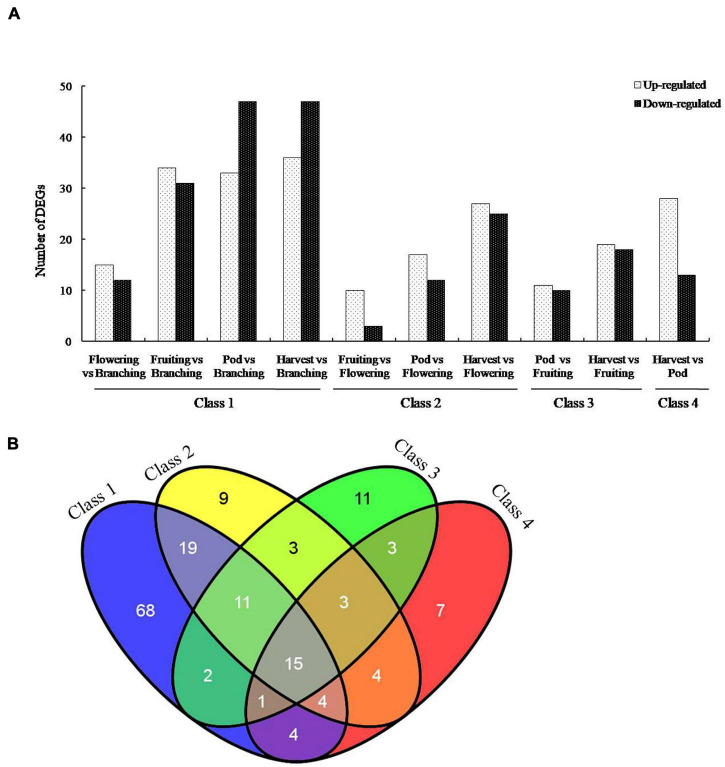
Genes differentially expressed in soybean nodules at five developmental periods of soybean. **(A)** Differentially expressed genes (DEGs) of *B. diazoefficiens* 113-2 between different nodule samples from five developmental periods of soybean. These DEGs were separated into two groups according to whether they were significantly up-regulated or down-regulated. Class 1, Flowering vs. Branching∪Fruiting vs. Branching∪Pod vs. Branching ∪ Harvest vs. Branching. Class 2, Fruiting vs. Flowering ∪Pod vs. Flowering∪ Harvest vs. Flowering. Class 3, Pod vs. Fruiting∪Harvest vs. Fruiting. Class 4, Harvest vs. Pod. **(B)** Venn diagrams showing the number of DEGs in each gene set for the four classes.

To screen the important genes of *B. diazoefficiens* 113-2 involved in nodule development and senescence, a false discovery rate (FDR) ≤ 0.001 and | log2 ratio| ≥ 1 were used to identify the DEGs in the ten Groups. The numbers of up-regulated and down-regulated DEGs in these ten Groups were shown in Figure 2A, and the ID information on these DEGs was provided in Supplementary Table 3. The Group (Harvest vs. Branching) possessed the highest number of DEGs (36 up-regulated, 47 down-regulated), followed by Pod vs. Branching Group (33 up-regulated, 47 down-regulated) and Fruiting vs. Branching Group (34 up-regulated, 31 down-regulated). Besides, Harvest vs. Flowering Group (27 up-regulated, 25 down-regulated), Harvest vs. Pod Group (28 up-regulated, 13 down-regulated) and Harvest vs. Fruiting Group (19 up-regulated, 18 down-regulated) also had relative high number of DEGs. There were relatively low DEG numbers among the three Groups (Fruiting vs. Flowering, Pod vs. Flowering and Pod vs. Fruiting).

According to different reference developmental stages N, above-mentioned ten Groups were divided into four Classes (Figure 2A). The number of DEGs consistently found in all four Classes was 15 and the total number of DEGs identified during the soybean nodule development was 164 (Figure 2B).

### DEG Annotation and Expression Pattern Analysis

The above-mentioned 164 DEGs encoded peptides with 41∼3105 amino acid residues, and their detail sequence information was shown in Supplementary Table 4. These DEGs were annotated to evaluate their potential functions. Among them, five DEGs-encoding nodulation proteins (NodG, NolG, NoeK, and NoeJ), nine DEGs-encoding Nif proteins (NifB, NifD, NifE, NifH, NifK, NifN, NifQ, NifW, and NifX), three DEGs-encoding Fix proteins (FixA, FixC, and FixK), three DEGs-encoding transcriptional regulator proteins and nine DEGs-encoding ABC transporter-related proteins were identified. Besides, 27 DEGs were involved in type-III secretion system (T3SS), eight DEGs located in membrane and many DEGs encoded various proteases (Supplementary Table 5).

**FIGURE 3:**
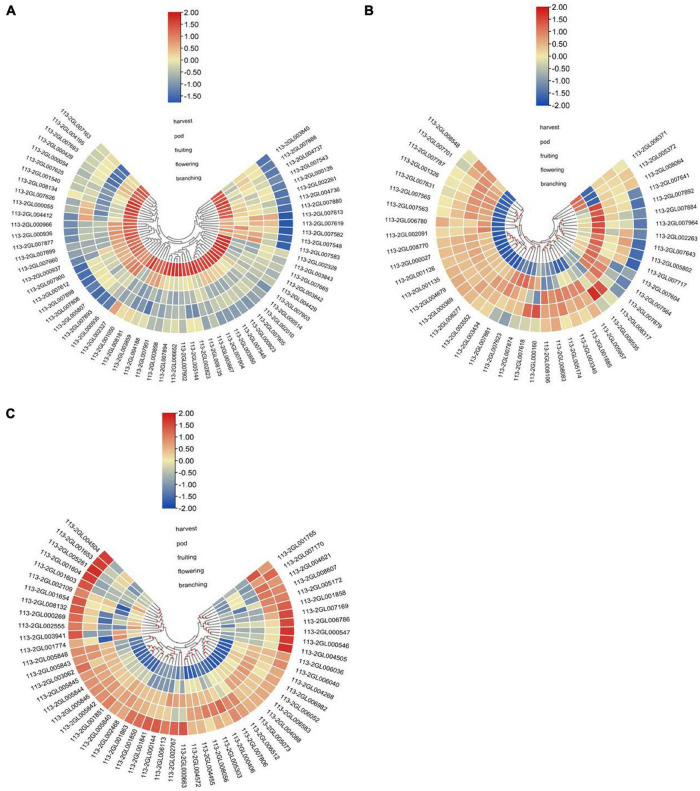
Heatmap plot showing the expression profile of the DEGs during nodule development and senescence. **(A)** Heatmap of DEGs mainly expressed in early nodule development. **(B)** Heatmap of DEGs maily expressed in nitrogen fixation progress. **(C)** Heatmap of DEGs mainly expressed in nodule senescence. These five nodule samples include the nodules at branching stage (1), flowering stage (2), fruiting stage (3), pod stage (4) and harvest stage (5), and the pheatmap packages in R were used to product this heatmap.

To investigate the expression profile of the 164 DEGs during nodule development and senescence, we analyzed the RNA-Seq results of these DEGs at above-mentioned five nodule samples, and the result showed that these DEGs had various expression patterns (Figure 3). 64 DEGs reached their peaks at branching stage_N or flowering stage_N, and most of them were down-regulated during nodule development and senescence (Figure 3A), indicating that these DEGs mainly play roles in the early stages of nodule development. 45 DEGs reached their peaks at flowering stage_N or fruiting stage_N, and had relative low expression at branching stage_N or harvest stage_N (Figure 3B), suggesting that these DEGs mainly participate in nitrogen fixation progress. About 55 DEGs reached their peaks at harvest stage_N or pod stage_N, and most of them were up-regulated during nodule development and senescence (Figure 3C), indicating that these DEGs mainly play roles in nodule senescence.

### DEG Functional Ontology and Kyoto Encyclopedia of Genes and Genomes Pathway Enrichment Analysis

**FIGURE 4:**
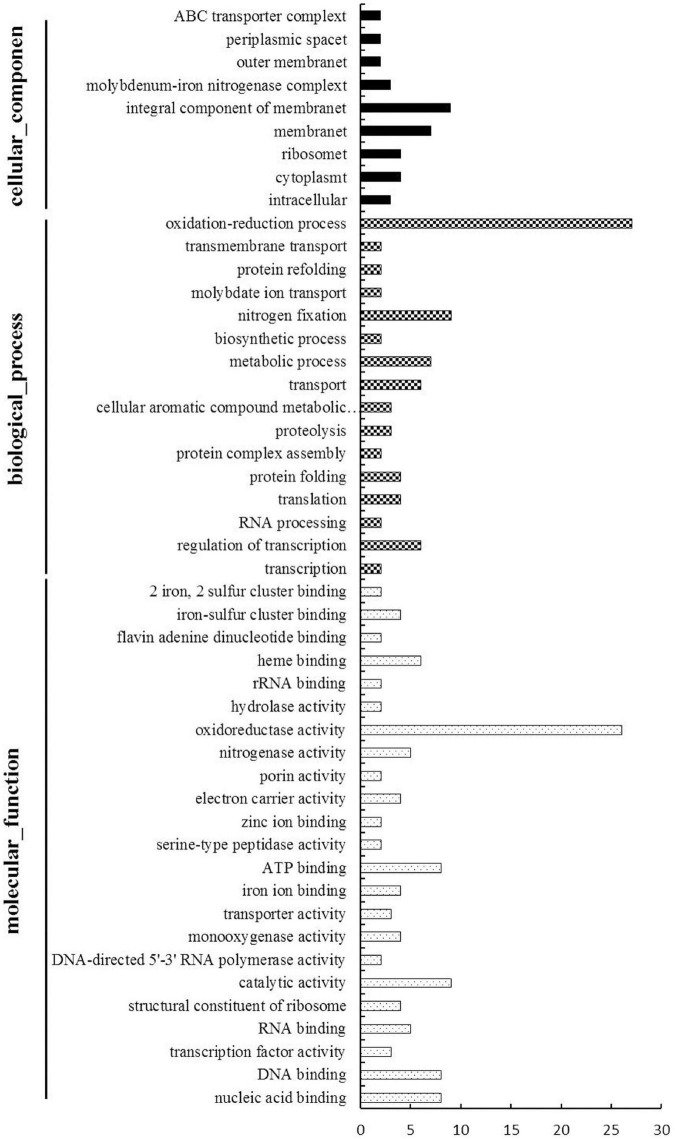
Gene Ontology-based functional annotation of DEGs of *B. diazoefficiens* 113-2 between different nodules from different developmental periods of soybean. Three GO domains (biological process, cellular components, and molecular function) are shown and the numbers of genes in each term were shown in histograms.

To evaluate the potential functions of the 164 DEGs, we used Gene Ontology (an internationally standardized gene function classification system) to classify these DEGs. Among these DEGs, 62 genes had no functional GO term, and a total of 150 GO terms were assigned for the rest genes, while about 2/3 (101 out of 150) functional GO terms were specific (only for one gene) (Supplementary Table 6). The rest49 Go terms were listed in Figure 4 and were divided into three categories: cellular components, biological process and molecular function. The cellular components mainly included integral component of membrane and membrane. The main biological processes of the DEGs were oxidation-reduction process, nitrogen fixation, metabolic process, transport and regulation of transcription. The molecular functions associated with the DEGs mainly focused on oxidoreductase activity, catalytic activity, ATP binding, nucleic acid binding and DNA binding.

**FIGURE 5:**
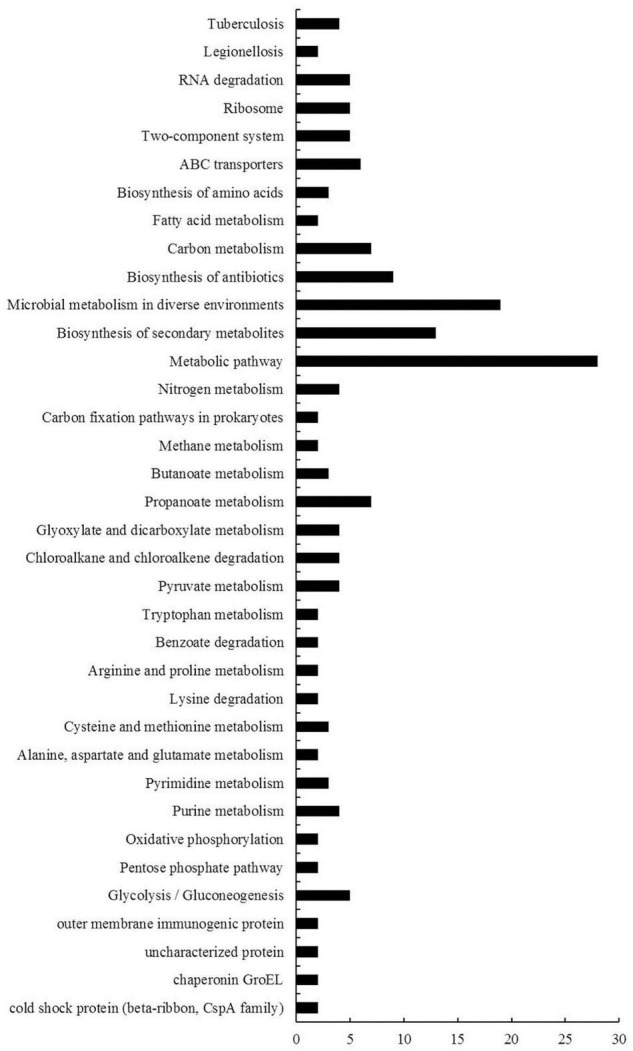
KEGG pathway enrichment analyses of DEGs of *B. diazoefficiens* 113-2 between different nodules from different developmental periods of soybean. 36 KEGG pathways were analyzed and the x- and y-axes represent pathway categories and the number of genes in each pathway, respectively.

KEGG is the major public database for pathway enrichment analysis (Kanehisa et al., 2016). In this report, we used KEGG database to classify these DEGs. Among these DEGs, 74 genes had no classified KEGG pathways, and a total of 149 KEGG pathway subgroups were assigned for the rest genes, while about 76% (113 out of 149) pathway subgroups were specific (only for one gene) (Supplementary Table 7). The other 36 pathway subgroups associated with the DEGs were shown in Figure 5. Most of these pathways were metabolic-related pathways. The pathways with the greatest numbers were metabolic pathway, followed by microbial metabolism in diverse environments, biosynthesis of secondary metabolites and biosynthesis of antibiotics.

### DEGs Associated With Nitrogen Metabolism, ABC Transporters and Two-Component System Pathways

**FIGURE 6:**
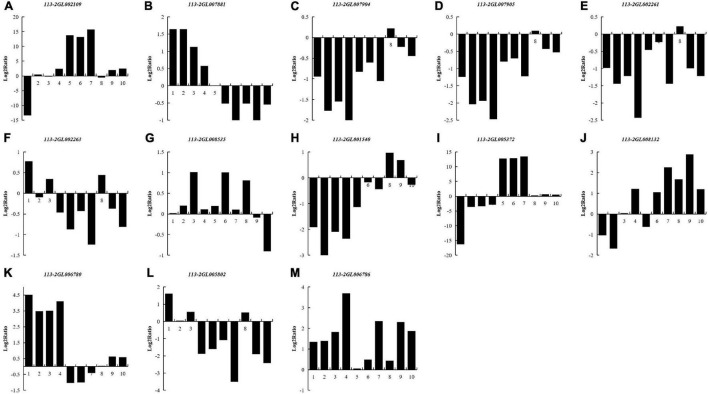
List of the DEGs associated with three pathways between nodules from different developmental periods of soybean. Nitrogen metabolism (Ko00910) **(A–D)**: NirK (113-2GL002109), nifDKH (113-2GL007881, 113-2GL007904 and 113-2GL007905); ABC transporters (Ko02010) **(E–J)**: modC (113-2GL002261), modA (113-2GL002263), pstS (113-2GL008535), gltI, aatJ (113-2GL001540), lptG (113-2GL005372), urtA (113-2GL008132); Two-component system (Ko02020) **(G,H,K–M)**: pstS (113-2GL008535), gltI, aatJ (113-2GL001540), ccoN (113-2GL006780), atoB (113-2GL005802) and fixK (113-2GL006786). 1, branching stage vs. flowering stage; 2, branching stage vs. fruiting stage; 3, branching stage vs. pod stage; 4, branching stage vs. harvest stage; 5, flowering stage vs. fruiting stage; 6, flowering stage vs. pod stage; 7, flowering stage vs. harvest stage; 8, fruiting stage vs. pod stage; 9, fruiting stage vs. harvest stage; 10, pod stage vs. harvest stage.

To investigate the regulation of the DEGs of 113-2 during nodule development and senescence, we analyzed nitrogen metabolism (K00910), ABC transporters (K02010) and Two-Component system (K02020) pathways (obtained by KEGG)^1^ in more detail (Figure 6). Two KEGG gene sets in K00910, seven in K02010 and six in K02020 were differentially expressed during nodule development and senescence. One gene that matched K00910 pathway gene *NirK* (*113-2GL002109*) was up-regulated in Harvest vs. Branching, Harvest vs. Flowering, Harvest vs. Fruiting and Harvest vs. Pod, suggesting that this gene has relative higher expression in harvest stage nodules and plays key roles in nodule senescence. Among the three DEGs that matched k02010 pathway gene *nifDKH*, *113-2GL007881* has different expression patterns with *113-2GL007904* or *113-2GL007905* in Flowering vs. Branching, Fruiting vs. Branching, Pod vs. Branching and Harvest vs. Branching groups, indicating that *nifDKH* gene may play diverse roles in nodulation and nodule development. For K02010 pathway gene sets, three genes that matched *modC*, *modA, gltI, and aatJ* were down-regulated in all of the detected groups, the other gene that matched *pstS* was up-regulated in Pod vs. Branching and Pod vs. Flowering groups, and the rest two genes *lptG* and *urtA* were down-regulated then up-regulated during nodule development and senescence, suggesting that they may have roles in both nodulation and nodule senescence. Three K02020 pathway gene sets (*pstS*, *gltI* and *aatJ*) were the same as that of K02010 pathway, and among the rest three gene sets, one DEG that matched *fixK* (*113-2GL006786*) was up-regulated in all of the detected groups, one gene that matched *ccoN* (*113-2GL006780*) was up-regulated in most of the detected groups and only down-regulated in Fruiting vs. Flowering group, while the rest one DEG that matched *atoB* (*113-2GL005802*) was down-regulated in most of the detected groups and only up-regulated in Flowering vs. Branching group.

### DEGs Encoding Nod and T3SS Secretion System-Related Proteins

**FIGURE 7:**
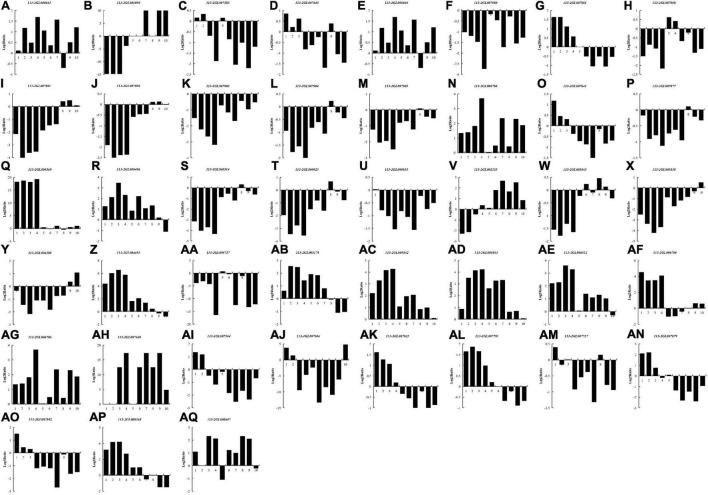
The Log2Ratio values of the DEG encoding Nod-related genes and T3SS secretion system-related genes during nodule development and senescence. *Nod* genes: **(A–E)**; *Nif* genes: **(F–M)**; *Fix* genes: **(N–P)**; T3SS-related genes: **(Q–AQ)**. 1, branching stage vs. flowering stage; 2, branching stage vs. fruiting stage; 3, branching stage vs. pod stage; 4, branching stage vs. harvest stage; 5, flowering stage vs. fruiting stage; 6, flowering stage vs. pod stage; 7, flowering stage vs. harvest stage; 8, fruiting stage vs. pod stage; 9, fruiting stage vs. harvest stage; 10, pod stage vs. harvest stage.

In most rhizobia, NFs, surface polysaccharides and secreted proteins are the main determinants of host specificity (Putnoky et al., 1988; Lorkiewicz, 1997; Li et al., 2014). To examine whether these three signaling molecules also play roles during nodule development and senescence, we explored DEGs that required for the biological synthesis of these signaling molecules between different nodules from different developmental periods of soybean, including five *Nod* genes, eight *nif* genes, three *fix* genes and 27 T3SS-related genes (Figure 7). 16 DEGs (two *Nod* genes, seven *nif* genes, one *fix* gene and six T3SS-related genes) were down-regulated in all of the detected groups, suggesting that these genes mainly play roles in nodule development rather than nodule senescence. Ten DEGs (one *Nod* gene, one *fix* gene and eight T3SS-related genes) were up-regulated in all of the detected groups, and most of these genes (except for *113-2GL007701*) have relative higher expression in pod stage nodules or harvest stage nodules, meaning that these genes may participate in regulating nodule senescence. Seven DEGs (*113-2GL007881*, *113-2GL007641*, *113-2GL007564*, *113-2GL007604*, *113-2GL007623*, *113-2GL007879*, and *113-2GL007892*) have highest expression in flowering stage nodules or fruiting stage nodules, reflecting that these genes may be critical to nitrogen fixation progress. Four DEGs (*113-2GL001050*, *113-2GL008064*, *113-2GL002555*, and *113-2GL004188*) were down-regulated then up-regulated during nodule development and senescence, suggesting that these genes may play roles in both in the early stages of nodule development and nodule senescence. Five genes (*113-2GL000406*, *113-2GL005174*, *113-2GL006780*, *113-2GL007717*, and *113-2GL008548*) have relative high expression in flowering, fruiting or pod stage nodules, indicating that they mainly play roles in nitrogen fixation progress. The rest one gene (*113-2GL007717*) was only down-regulated in Harvest/Flowering group.

### Orthologs of the DEGs

To further study the roles of these rhizobial DEGs during nodule development and senescence, we identified their orthologs in 113-2 itself, USDA110, *Mesorhizobium loti* MAFF303099, *Mesorhizobium huakuii* 7653R and *Sinorhizobium meliloti* 2011. Four homologous pairs (*113-2GL000406*/*113-2GL001850*, *113-2GL004736*/*113-2GL006371*, 113-2GL004195/113-2GL002328, and 113-2GL003941/113-2GL001604) were existed in these 164 DEGs. 16 genes have no orthologs, and most of them have no COG functions (Supplementary Table 8), indicating that these DEGs were mainly strain-specific. 84 DEGs have only orthologs in USDA110, suggesting that these DEGs were mainly species-specific or host specific, and among these DEGs, about 13.1% (11 out of 84) have orthologs in 113-2 itself and more than one orthologs in USDA110 (Table 2). Among the rest 62 DEGs, 35 DEGs have one or more orthologs in all of the five rhizobia, 11 DEGs have orthologs in four rhizobia and 16 DEGs have orthologs in three rhizobial, reflecting that not all of these nodule development and senescence-related DEGs are core genes among these four rhizobial (Table 3). Among the core 35 DEGs, about 60% (21 out 35) were mainly expressed in the early stages of nodule development, six DEGs showed highest expression in the progress of nitrogen fixation, and eight DEGs may play critical roles in nodule senescence, indicating that these core genes have diverse roles during nodule development and senescence (Table 3). Among these four rhizobia, 113-2 or USDA110 and *M. loti* MAFF303099 can form determinate nodules with soybean and *Lotus*, respectively, and nine DEGs of 113-2 have orthologs only in USDA110 and *M. loti* MAFF303099 (Table 3). Besides, we selected four gene groups with more orthologs to perform phylogeny analyses, and the results revealed closer phylogenetic relationships between the two strains in the same genus (Supplementary Figures 4–7).

**TABLE 2:** Analysis of species-specific or host specific DEGs.

DEGs	113-2	USDA110	COG annotion
*113-2GL000027*		*bll0805*	CubicO group peptidase, beta-lactamase class C family
*113-2GL000144*		*blr0694*	Serine protease, subtilase family
*113-2GL000369*		*blr0497*	–
*113-2GL000546*		*bll0333*	Glucose dehydrogenase
*113-2GL000547*		*bll0332*	Cytochrome c, mono- and diheme variants
*113-2GL000923*	*113-2GL007956*	*blr1693,bll8244*	–
*113-2GL000935*		*bll8232*	L-lysine 2,3-aminomutase (EF-P beta-lysylation pathway)
*113-2GL000936*		*bll8231*	Gamma-glutamyltranspeptidase
*113-2GL000937*		*bll8230*	–
*113-2GL000966*		*bll8195*	Retron-type reverse transcriptase
*113-2GL001135*		*bll7952*	DNA-binding beta-propeller fold protein YncE
*113-2GL001326*		*bll7787*	–
*113-2GL001540*	*113-2GL005055,113-2GL005658*	*bll4384,bll7600,blr3849*	ABC-type amino acid transport/signal transduction system, periplasmic component/domain
*113-2GL001603*		*bll7540*	Fe-S oxidoreductase
*113-2GL001604*	*113-2GL003941*	*bll7539,blr5311*	–
*113-2GL001653*		*bll7492*	Predicted DNA-binding protein, contains Ribbon-helix-helix (RHH) domain
*113-2GL001765*		*bll7395*	Uncharacterized protein
*113-2GL001774*		*bll7385*	Cellulose biosynthesis protein BcsQ
*113-2GL001885*		*blr7289*	Flavin-dependent oxidoreductase, luciferase family
*113-2GL002261*		*blr6953*	ABC-type molybdate transport system, ATPase component
*113-2GL002263*		*blr6951*	ABC-type molybdate transport system, periplasmic component
*113-2GL002327*		*blr6889*	DNA-binding transcriptional regulator, MarR family
*113-2GL002328*	*113-2GL004195, 113-2GL004285*	*bll5076,bll4983,bll6888*	–
*113-2GL002767*		*bll6479*	ABC-type branched-chain amino acid transport system, periplasmic component
*113-2GL002823*		*bll6433*	–
*113-2GL003062*		*bll6129*	Uncharacterized protein
*113-2GL004088*		*bll5176*	–
*113-2GL004268*		*bsl5002*	–
*113-2GL004412*	*113-2GL004612, 113-2GL002719*,	*blr4994,blr5174,bll6524*,	Opacity protein and related surface antigens
	*113-2GL004272, 113-2GL004615*,	*blr4699,bll4867,blr4701*,	
	*113-2GL001425, 113-2GL004090*	*blr7695*	
*113-2GL004429*	*113-2GL005811*	*bll4853,blr3712*	Outer membrane protein assembly factor BamA
*113-2GL004621*		*bsr4694*	–
*113-2GL004679*		*blr4646*	CBS domain
*113-2GL004737*		*bll4594*	Fatty-acid desaturase
*113-2GL005073*		*bll4367*	Glyoxylase or a related metal-dependent hydrolase, beta-lactamase superfamily II
*113-2GL005144*		*bll4305*	Ribonuclease G or E
*113-2GL005174*		*bll4278*	–
*113-2GL005281*		*blr4186*	Glyoxylase or a related metal-dependent hydrolase, beta-lactamase superfamily II
*113-2GL005303*		*bsl4167*	–
*113-2GL005372*		*bll4106*	Lipopolysaccharide export LptBFGC system, permease protein LptF
*113-2GL005840*		*blr3683*	Chaperonin GroEL (HSP60 family)
*113-2GL005842*		*blr3681*	Predicted metal-dependent hydrolase, TIM-barrel fold
*113-2GL005843*		*blr3680*	–
*113-2GL005844*		*blr3679*	–
*113-2GL005845*		*blr3678*	Ferredoxin-NADP reductase
*113-2GL005846*		*blr3677*	–
*113-2GL005848*		*blr3675*	D-arabinose 1-dehydrogenase, Zn-dependent alcohol dehydrogenase family
*113-2GL006036*		*blr3477*	Aspartate/methionine/tyrosine aminotransferase
*113-2GL006040*		*blr3474*	Arginine/lysine/ornithine decarboxylase
*113-2GL006052*		*blr3464*	Uncharacterized protein
*113-2GL006113*		*blr3404*	Heme-degrading monooxygenase HmoA and related ABM domain proteins
*113-2GL006583*		*bll2956*	Sugar lactone lactonase YvrE
*113-2GL007613*	*113-2GL000934, 113-2GL007832*,	*blr2165,blr7550,blr1829*,	Transposase
	*113-2GL007909, 113-2GL000934*,	*blr1911,blr4868,blr1716*,	
	*113-2GL007752, 113-2GL007853*,	*blr1702,bll4642,blr1740*,	
	*113-2GL007932, 113-2GL004410*	*blr8233,bll8293,blr2076*,	
		*blr1807*	
*113-2GL006652*		*blr2887*	Uncharacterized protein
*113-2GL006786*	*113-2GL007581, 113-2GL006786*,	*bll3466,bll2109,bll2757*	cAMP-binding domain of CRP or a regulatory subunit of cAMP-dependent protein kinases
	*113-2GL006050*		
*113-2GL006982*		*bll2590*	Nucleotide-binding universal stress protein, UspA family
*113-2GL007163*		*bll2460*	Outer membrane receptor for ferrienterochelin and colicins
*113-2GL007170*		*blr2455*	Isocitrate lyase
*113-2GL007564*		*blr2132*	–
*113-2GL007565*		*blr2131*	Lysine/ornithine N-monooxygenase
*113-2GL007582*		*blr2108*	Non-ribosomal peptide synthetase component F
*113-2GL007583*		*blr2106*	Mannose-6-phosphate isomerase, cupin superfamily
*113-2GL007612*		*blr2077*	O-acetylhomoserine/O-acetylserine sulfhydrylase, pyridoxal phosphate-dependent
*113-2GL007619*		*bll2067*	–
*113-2GL007623*		*bll2063*	Acyl-CoA dehydrogenase related to the alkylation response protein AidB
*113-2GL007660*		*bll2012*	Opacity protein and related surface antigens
*113-2GL007665*		*bll2003*	Carbohydrate-selective porin OprB
*113-2GL007693*		*blr1971*	Dipeptidyl aminopeptidase/acylaminoacyl peptidase
*113-2GL007717*		*bll1944*	Opacity protein and related surface antigens
*113-2GL007787*	*113-2GL000704*	*bll0184,blr1879*	DnaJ-class molecular chaperone with C-terminal Zn finger domain
*113-2GL007806*	*113-2GL001935, 113-2GL001936*	*blr7243,blr1853,blr7242*	Cytochrome P450
113-2GL007808		blr1851	Uncharacterized protein YjbI, contains pentapeptide repeats
113-2GL007874		bll1777	Alkyl hydroperoxide reductase subunit AhpC (peroxiredoxin)
113-2GL007880		blr1770	–
113-2GL007884	113-2GL008289	blr1311,bll1766	Outer membrane protein W
113-2GL007893		bll1754	–
113-2GL007894		blr1753	Ferredoxin subunit of nitrite reductase or a ring-hydroxylating dioxygenase
113-2GL007900		blr1748	–
113-2GL007901		blr1747	Predicted Fe-Mo cluster-binding protein, NifX family
113-2GL007903		blr1745	Nitrogenase molybdenum-iron protein, alpha and beta chains
113-2GL008083		blr1496	Uncharacterized conserved protein YjbJ, UPF0337 family
113-2GL008134		bll1447	Superfamily II DNA and RNA helicase
113-2GL008317		bll1285	–
113-2GL008548		blr1081	1,2-phenylacetyl-CoA epoxidase, catalytic subunit
113-2GL008607	113-2GL006492	bll1028, blr3042	DNA-directed RNA polymerase specialized sigma subunit, sigma24 family

**TABLE 3:** Orthologs of the selected DEGs in 113-2, USDA110, *M. loti* MAFF303099, *M. huakuii* 7653R, and *S. meliloti* 2011.

DEGs	Orthologs of the DEGs					Expression in soybean symbiosis
	113-2	USDA110	MAFF303099	7653R	SM2011	(according to Figure 3)
*113-2GL000054*		*bll0779*	*MAFF_RS22785*	*MCHK_RS30795*	*SM2011_RS15560*	Early stages of nodule development
*113-2GL000055*		*blr0778*	*MAFF_RS28315*	*MCHK_RS02430*	*SM2011_RS06535*	Early stages of nodule development
*113-2GL000126*		*bll0710*	*MAFF_RS20750*	*MCHK_RS28780*	*SM2011_RS15760*	Early stages of nodule development
*113-2GL000160*		*blr0678*	*MAFF_RS19575*	*MCHK_RS27375*	*SM2011_RS15250*	Nodule senescence
*113-2GL000269*		*blr0573*	*MAFF_RS17030*	*MCHK_RS25150*	*SM2011_RS31340*	Nodule senescence
*113-2GL000406*	*113-2GL001850*	*bll7322,blr0462*	*MAFF_RS17955*	*MCHK_RS26065*	*SM2011_RS30815*	Nodule senescence
*113-2GL000429*		*bll0442*	*MAFF_RS16950*	*MCHK_RS25060*	*SM2011_RS29850*	Early stages of nodule development
*113-2GL000514*	*113-2GL002082*	*bsr7117, blr0365*	*MAFF_RS13260*	*MCHK_RS21570*	*SM2011_RS24865*	Early stages of nodule development
*113-2GL000663*	*113-2GL005801*	*bll0225,blr3725*	*MAFF_RS16145*		*SM2011_RS31010*	Nodule senescence
*113-2GL001050*		*blr8272,bll8126*			*SM2011_RS09965*	Early stages of nodule development
*113-2GL001126*	*113-2GL004687*	*blr4637,blr7961*		*MCHK_RS12390*,	*SM2011_RS03045*	Nitrogen fixation
				*MCHK_RS18535*		
*113-2GL001654*		*blr7491*		*MCHK_RS11600*		Nodule senescence
*113-2GL001841*		*blr7327*	*MAFF_RS27500*			Nodule senescence
*113-2GL001851*		*blr7321*	*MAFF_RS27480*		*SM2011_RS04580*	Nodule senescence
*113-2GL001863*		*bll7310*	*MAFF_RS27520*	*MCHK_RS18460*	*SM2011_RS04570*,	Nodule senescence
					*SM2011_RS01895*	
*113-2GL002091*		*bsl7109*			*SM2011_RS33980*	Nitrogen fixation
*113-2GL002109*		*blr7089*		*MCHK_RS17580*	*SM2011_RS03405*	Nodule senescence
*113-2GL002468*		*bll6756*	*MAFF_RS00585*		*SM2011_RS21450*	Nodule senescence
*113-2GL003340*	*113-2GL001478*	*bll7648,bll5866*	*MAFF_RS18965*	*MCHK_RS27010*		Nitrogen fixation
*113-2GL003434*		*blr5774*		*MCHK_RS17320*		Nitrogen fixation
*113-2GL003840*		*bll5412*	*MAFF_RS02150*	*MCHK_RS09810*	*SM2011_RS21190*	Early stages of nodule development
*113-2GL003842*		*bll5410*	*MAFF_RS02160*	*MCHK_RS09820*	*SM2011_RS21200*	Early stages of nodule development
*113-2GL003843*		*bll5409*	*MAFF_RS02165*	*MCHK_RS09825*	*SM2011_RS21205*	Early stages of nodule development
*113-2GL003850*		*bll5402*	*MAFF_RS02200*,		*SM2011_RS21230*,	Early stages of nodule development
			*MAFF_RS02105*		*SM2011_RS21150*	
*113-2GL003858*		*bll5394*	*MAFF_RS02240*	*MCHK_RS09900*	*SM2011_RS21270*	Early stages of nodule development
*113-2GL003859*		*bll5393*	*MAFF_RS02245*	*MCHK_RS09905*	*SM2011_RS21275*	Early stages of nodule development
*113-2GL003867*		*bll5385*	*MAFF_RS02285*	*MCHK_RS09945*	*SM2011_RS21315*	Early stages of nodule development
*113-2GL004505*		*bll4784*	*MAFF_RS27120*	*MCHK_RS01750*	*SM2011_RS26345*	Nodule senescence
*113-2GL004572*	*113-2GL005733*	*blr4723,blr3787*		*MCHK_RS07395*		Nodule senescence
*113-2GL004736*	*113-2GL001679*,	*bsl4595,bsr7468*,	*MAFF_RS33280*,	*MCHK_RS19875*,	*SM2011_RS02010*,	Early stages of nodule development
	*113-2GL006371*,	*bsl4595,bsl1445*,	*MAFF_RS00310*,	*MCHK_RS07180*,	*SM2011_RS24070*,	
	*113-2GL008136*	*bsr3154*	*MAFF_RS11065*,	*MCHK_RS19840*,	*SM2011_RS00515*,	
			*MAFF_RS11510*	*MCHK_RS32390*	*SM2011_RS26245*	
			*MAFF_RS25580*,	*MCHK_RS19440*,	*SM2011_RS25125*,	
			*MAFF_RS11485*	*MCHK_RS31800*	*SM2011_RS24855*	
*113-2GL005802*		*bll0226,blr3724*	*MAFF_RS16140*	*MCHK_RS24370*,	*SM2011_RS31015*	Nitrogen fixation
				*MCHK_RS02525*		
*113-2GL005957*		*blr3566*	*MAFF_RS21725*	*MCHK_RS29825*		Nitrogen fixation
*113-2GL006512*		*bll3022*	*MAFF_RS15360*	*MCHK_RS23560*		Nodule senescence
*113-2GL006780*		*blr2763*	*MAFF_RS27090*,	*MCHK_RS01720*	*SM2011_RS02090*,	Nitrogen fixation
			*MAFF_RS26180*		*SM2011_RS01660*,	
					*SM2011_RS03325*	
*113-2GL007543*		*blr2149*	*MAFF_RS26000*			Early stages of nodule development
*113-2GL007545*		*blr2147*	*MAFF_RS25985*			Early stages of nodule development
*113-2GL007548*		*blr2145*	*MAFF_RS25975*			Nitrogen fixation
*113-2GL007604*	*113-2GL007555*,	*bll3527,bll1900*,	*MAFF_RS25425*	*MCHK_RS31900*	*SM2011_RS30325*	Nitrogen fixation
	*113-2GL003457*,	*bll5000,blr1706*				
	*113-2GL008356*,	*bll1997*				
	*113-2GL007792*,					
	*113-2GL004069*,					
	*113-2GL000973*,					
	*113-2GL007670*,					
	*113-2GL007896*,					
	*113-2GL007767*,					
	*113-2GL007630*,					
	*113-2GL007942*,					
	*113-2GL004713*,					
	*113-2GL007823*					
*113-2GL007618*		*blr2068*	*MAFF_RS25050*			Nodule senescence
*113-2GL007625*	*113-2GL002237*,	*blr5226,blr6978*,	*MAFF_RS33345*,	*MCHK_RS17610*,	*SM2011_RS18355*,	Early stages of nodule development
	*113-2GL007625*,	*blr5625,blr5226*,	*MAFF_RS09905*,	*MCHK_RS07245*	*SM2011_RS00340*,	
	*113-2GL001613*,	*bll2060,bsr7532*	*MAFF_RS10535*,		*SM2011_RS02030*,	
	*113-2GL004032*,		*MAFF_RS36295*,		*SM2011_RS11615*	
	*113-2GL003601*		*MAFF_RS23815*			
*113-2GL007626*	*113-2GL001611*,	*blr5626,bll2059*,	*MAFF_RS23810*,	*MCHK_RS07240*	*SM2011_RS11610*,	Early stages of nodule development
	*113-2GL004031*,	*blr6979,blr5227*,	*MAFF_RS36300*,		*SM2011_RS20400*,	
	*113-2GL003600*,	*blr4635,blr7533*	*MAFF_RS33340*,		*SM2011_RS18350*,	
	*113-2GL002236*,		*MAFF_RS10540*		*SM2011_RS02025*	
	*113-2GL004689*					
*113-2GL007641*		*blr2038*	*MAFF_RS24010*	*MCHK_RS32450*	*SM2011_RS02275*	Nitrogen fixation
*113-2GL007643*	*113-2GL006940*	*bll2623,blr2036*	*MAFF_RS13735*	*MCHK_RS22150*	*SM2011_RS04845*	Nitrogen fixation
*113-2GL007877*		*blr1774*	*MAFF_RS24000*	*MCHK_RS32460*	*SM2011_RS02265*	Early stages of nodule development
*113-2GL007879*		*blr1771*	*MAFF_RS24015*			Nitrogen fixation
*113-2GL007881*		*blr1769*	*MAFF_RS24195*	*MCHK_RS32325*	*SM2011_RS02280*	Nitrogen fixation
*113-2GL007888*		*blr1759*	*MAFF_RS23985*	*MCHK_RS32485*	*SM2011_RS02250*	Early stages of nodule development
*113-2GL007892*		*blr1755*	*MAFF_RS24355*			Nitrogen fixation
*113-2GL007898*		*bsr1750*	*MAFF_RS24040*,			Early stages of nodule development
			*MAFF_RS24300*			
*113-2GL007899*		*bsr1749*	*MAFF_RS24035*,			Early stages of nodule development
			*MAFF_RS24295*			
*113-2GL007902*		*blr1746*	*MAFF_RS24215*	*MCHK_RS32305*	*SM2011_RS02420*	Early stages of nodule development
*113-2GL007904*		*blr1744*	*MAFF_RS24205*	*MCHK_RS32315*	*SM2011_RS02290*	Early stages of nodule development
*113-2GL007905*		*blr1743*	*MAFF_RS24200*	*MCHK_RS32320*	*SM2011_RS02285*	Early stages of nodule development
*113-2GL007964*	*113-2GL005342*	*blr1686,blr4134*			*SM2011_RS08430*	Nitrogen fixation
*113-2GL008056*		*bll1523*	*MAFF_RS15765*	*MCHK_RS33030*,	*SM2011_RS28455*	Nodule senescence
				*MCHK_RS23965*		
*113-2GL008064*		*blr1516*			*SM2011_RS15300*	Early stages of nodule development
*113-2GL008106*		*bsl1473*	*MAFF_RS37185*		*SM2011_RS11135*	Nitrogen fixation
*113-2GL008132*	*113-2GL003149*	*blr1448,blr6053*	*MAFF_RS04190*	*MCHK_RS11590*	*SM2011_RS14035*	Nodule senescence
*113-2GL008135*		*bsl1446*	*MAFF_RS26365*	*MCHK_RS00670*	*SM2011_RS17430*	Early stages of nodule development
*113-2GL008535*		*blr1091*	*MAFF_RS15660*	*MCHK_RS23865*		Nodule senescence

Our previous study has indicated that 113-2 has an extremely close phylogenetic relationship to USDA110 (Li et al., 2020). An interspecies protein interactome was constructed and 290 USDA110 proteins associated with soybean symbiosis were provided (Zhang et al., 2018). In this report, about 203 USDA110 proteins were searched from the orthologs of the DEGs (Tables 2, 3). We compared these two USDA110 protein groups and discovered six same proteins (bll7322, blr1971, blr1091, blr1516, blr0462, and bll7600), which were detected to be expressed in rhizoidal of root nodules during symbiosis in previous studies (Pessi et al., 2007; Delmotte et al., 2010; Čuklina et al., 2016; Zhang et al., 2018). Then we predict the interaction proteins of the six DEGs (orthologs of the six USDA110 proteins) according to the resources from the constructed protein interactome (Zhang et al., 2018), and the results were showed in Table 4. These rhizoidal proteins have more than two predicted interacting proteins in soybean, especially for bll7322 or 113-2GL000406 or 113-2GL001850, and blr1971 or 113-2GL007693; they have 21 and 25 predicted interacting proteins in soybean, respectively.

**TABLE 4:** The prediction of the interaction proteins of the selected DEGs.

DEGs	Orthologs in USDA110	References for orthologs	Potencial interaction proteins		
			Soybean	USDA110	113-2
113-2GL000406	bll7322	Zhang et al., 2018	Glyma.01G003800	bll7785	113-2GL002891
113-2GL001850			Glyma.02G017800		113-2GL001328
			Glyma.02G147200		
			Glyma.02G222300		
			Glyma.03G164400		
			Glyma.03G251900		
			Glyma.04G080800		
			Glyma.04G082000		
			Glyma.04G194700		
			Glyma.04G226700		
			Glyma.06G081900		
			Glyma.06G082500		
			Glyma.06G171200		
			Glyma.09G273200		
			Glyma.10G018200		
			Glyma.12G045400		
			Glyma.12G167500		
			Glyma.16G043900		
			Glyma.18G284300		
			Glyma.19G222600		
			Glyma.20G249300		
	blr0462	Pessi et al., 2007	Glyma.08G152600		
		Delmotte et al., 2010	Glyma.15G272000		
		Čuklina et al., 2016			
		Zhang et al., 2018			
113-2GL007693	blr1971	Pessi et al., 2007	Glyma.01G118900	blr1971	113-2GL007693
		Delmotte et al., 2010	Glyma.02G155300	bll5750	113-2GL003467
		Čuklina et al., 2016	Glyma.02G208700		
		Zhang et al., 2018	Glyma.03G004900		
			Glyma.03G195400		
			Glyma.04G007400		
			Glyma.04G081100		
			Glyma.05G204000		
			Glyma.07G079700		
			Glyma.07G173100		
			Glyma.08G067400		
			Glyma.09G278600		
			Glyma.10G280000		
			Glyma.11G102100		
			Glyma.11G237400		
			Glyma.14G176900		
			Glyma.14G206300		
			Glyma.15G058400		
			Glyma.16G117600		
			Glyma.18G056100		
			Glyma.18G060600		
			Glyma.18G210100		
			Glyma.19G173400		
			Glyma.19G205200		
			Glyma.20G005900		
113-2GL008535	blr1091	Pessi et al., 2007	Glyma.02G294900	blr1148	113-2GL008473
		Delmotte et al., 2010	Glyma.14G018700	blr3906	113-2GL005583
		Čuklina et al., 2016	Glyma.14G161800	blr4307	113-2GL005142
		Zhang et al., 2018	Glyma.14G162000	bll4945	113-2GL004326
113-2GL008064	blr1516	Pessi et al., 2007	Glyma.05G033800	blr1516	113-2GL008064
		Delmotte et al., 2010	Glyma.14G145000	bll3640	113-2GL005884
		Čuklina et al., 2016		blr4473	113-2GL004883
		Zhang et al., 2018		bll4736	113-2GL004557
				bll5710	113-2GL003508
113-2GL001540	bll7600	Delmotte et al., 2010	Glyma.17G228800	bll7600	113-2GL001540
		Čuklina et al., 2016	Glyma.18G277300		
		Zhang et al., 2018			

## Discussion

Symbiotic nitrogen fixation system is of great significance in agriculture and ecology, and nodule development and senescence directly affects nitrogen fixation efficiency. Nodule development and senescence involves a programmed series of molecular events that are carried out synchronously by both legumes and rhizobia (Vasse et al., 1990; Cermola et al., 2000; Oldroyd et al., 2011; Roux et al., 2014; Alemneh et al., 2020). The nitrogen fixation characteristics of five soybean developmental stages (Branching stage, flowering stage, fruiting stage, pod stage and harvest stage) have been well studied (Fehr et al., 1971), the expression of nodule genes at these developmental stages was assessed quantitatively using RNA-Seq, and the important DEGs of soybean that associated with nodule development and senescence were identified in our previous study (Yuan et al., 2017). In the present study, we firstly investigated the expression of rhizobial genes in the nodule samples from above-mentioned five developmental stages of soybean by using our previous RNA-Seq data (Yuan et al., 2017)and identified 164 important rhizobial DEGs associated with nodule development and senescence.

### The Gene Expression of *B. diazoefficiens* 113-2 at Different Nodule Samples From Different Soybean Developmental Stages

Previous comparative transcriptomic analyses have explored the gene expression patterns of rhizobia during chemoautotrophic growth (Franck et al., 2008) or symbiotic non-growth (Vercruysse et al., 2011), under different stresses (Ampe et al., 2003; Chang et al., 2007; Cytryn et al., 2007), in determinate nodules and indeterminate nodules (Li Y. et al., 2013), and in different zones of the indeterminate nodule (Roux et al., 2014). For the bacteroids in soybean symbiosis, several transcriptomic analyses of bacteroids focused on symbiotic gene region (Hauser et al., 2006) and single time point during optimal symbiotic functioning (Chang et al., 2007; Pessi et al., 2007; Franck et al., 2014). It is worth noting that a multiple time-point microarray study in *B. diazoefficiens* bacteroids isolated from soybean nodules from nodulation to nodule senescence, which revealed a shift gene expression patterns of *B. diazoefficiens* during symbiotic process, especially in nodule senescence (Franck et al., 2018). In this report, we also performed a multiple time-point transcriptomic ananlysis, the difference is that the RNA samples were collected from different nodule samples from five important developmental stages of soybean (Yuan et al., 2017). We firstly investigated the expression of rhizobial genes in symbiosis at above-mentioned five developmental stages of soybean, which should shed new light on the molecular mechanisms of nitrogen fixation during the growth and development of soybean.

In the five soybean nodule samples, about 25.38% (2234 out of 8801) of *B. diazoefficiens* 113-2 genes were not detected, indicating that these genes may not participate in symbiotic progress. The pod stage nodules possessed the highest number of genes with meaningful fpkm values (>1), suggesting the initiation of a series of new processes, which might be associated with nodule senescence. The correlation analysis of the gene expressions in the five nodule samples showed that the expression of *B. diazoefficiens* 113-2 genes at Branching stage or harvest stage has relative lower correlation with the other soybean development stages, but high correlations were existed among flowering stage, fruiting stage and pod stage (Figure 1B), indicating that similar gene expression patterns of *B. diazoefficiens* 113-2 were found during optimal symbiotic functioning, but different expression patterns were existed among early nodule development, nitrogen fixation progress and nodule senescence. These results also revealed a shift gene expression pattern of *B. diazoefficiens* 113-2 in symbiosis during the growth and development of soybean.

### DEGs of *B. diazoefficiens* 113-2 Associated With Nodule Development and Senescence

Previous study showed that > 800 USDA110 genes were up- or down-regulated at each time point of soybean nodule development (Franck et al., 2018), while in this report, the highest number of DEGs at the ten groups was 83 (Figure 2A). The major reason may be that we used our previous soybean nodule RNA-Seq data, in which the RNA samples were mixed with both soybean RNA and *B. diazoefficiens* 113-2 RNA (Yuan et al., 2017). Our main purpose is to screen the important DEGs that can interact with host and are mainly involved in nodule development and senescence. The gene expression of soil rhizobia inside of the host was different from free live state (Vercruysse et al., 2011), so we did not isolate *B. diazoefficiens* 113-2 from the nodule samples.

Lots of important rhizobial genes have been showed to participate in nodule development and senescence (Voroshilova et al., 2001; Tittabutr et al., 2015; Franck et al., 2018). Recently, we sequenced the genome of *B. diazoefficiens* 113-2 (Li et al., 2020) and identified 164 DEGs of *B. diazoefficiens* 113-2 during the soybean nodule development in this study. Among these DEGs, 49 DEGs had both no functional GO term and no classified KEGG pathway (Supplementary Tables 6, 7), and about 13 DEGs were not clearly annotated (Supplementary Table 5), meaning that we identified many novel and uncharacterized genes associated with nodule development and senescence. About 62.2% (102 out of 164) of the DEGs have no orthologs in *M. loti* MAFF303099, *M. huakuii* 7653R and *S. meliloti*2011, and only 35 DEGs have orthologs in all of the three rhizobia (Table 3), indicating that most of the identified genes associated with nodule development and senescence are not core genes among the rhizobia genomes. Besides, *B. diazoefficiens* 113-2 and *M. loti* MAFF303099 can form determinate nodules with soybean and *Lotus*, respectively, but only nine DEGs of 113-2 have orthologs only in *M. loti* MAFF303099 (Table 3), suggesting that the nodule development and senescence in determinate nodules or indeterminate nodules was also mainly determined by host legume plants, which is similar to our previous study (Li et al., 2020).

### The Potential Roles of Host Specificity-Related DEGs in Nodule Development and Senescence

In most rhizobia, the genes that affect the biosynthesis of nodulation factors, secretion system and surface polysaccharides are often play critical roles in host specificity (Horvath et al., 1986; Philip-Hollingsworth et al., 1989; Wang et al., 2014). In this report, five *Nod* genes, eight *nif* genes, three *fix* genes and 27 T3SS-related genes (no genes associated with surface polysaccharides biosynthesis) were identified (Figure 7). The *Nod* genes primarily played roles in nodulation (Moulin et al., 2004), and among the identified five *Nod* genes, only *Noek* (*113-2GL001050*) and *NolG* (*113-2GL008064*) may play roles in the early state of nodule development. *NoeJ* (*113-2GL007583*) and *NolG* (*113-2GL007643*) may participate in nitrogen fixation progress and *NodG* (*113-2000663*) may have roles in nodule senescence. The *nif* and *fix* genes are crucial for nitrogen fixation progress (Fischer and Hennecke, 1984; Hennecke, 1990). NifH, NifD, NifK, NifE, and NifN proteins are the core components of nitrogenase (Raymond et al., 2004), and the *FixABCX* genes were found to encode a membrane complex participating in electron transfer to nitrogenase (Earl et al., 1987; Edgren and Nordlund, 2004). The two main regulatory cascades that can be found in rhizobia are the RpoN−NifA and the oxygen−responsive two−component FixL−FixJ system, together with FixK (Lindström and Mousavi, 2019), which was found to regulate positively and negatively nitrogen fixation genes in *Rhizobium meliloti* (Batut et al., 1989). Among the identified eight *Nif* genes, only *NifH* (*113-2GL007881*) and *NifW* (*113-2GL007879*) reached the expression peaks at flowering stage_N or fruiting stage_N, the rest six *nif* genes *NifB* (*113-2GL007888*), *NifD/K* (*113-2GL007904*, *113-2GL007095*), *NifE* (*113-2-GL007903*), *NifN* (*113-2GL007902*), *NifQ* (*113-2007880*), and *NifX* (*113-2GL007901*) may mainly involved in regulating the early nodule development. For the three *fix* genes, *FixA* (*113-2GL007641*) and *FixC* (*113-2GL007877*) had relative higher expression at branching stage_N and flower stage_N, implying that they may mainly play roles in nitrogen fixation progress, while *FixK* (*113-2GL006786*) may mainly have roles in nodule senescence. The rhizobia T3SS and its secreted effectors has been reported to modulate nodulation and host range (Okazaki et al., 2013). For the 27 T3SS-related genes, only seven DEGs had relative higher expression at branching stage_N, and the rest 20 genes may participate in nitrogen fixation progress or nodule senescence, meaning that the T3SS-related genes have roles in the whole nodule development and senescence rather than only in nodulation, which is similar to our previous study (Yuan et al., 2017). Together, these results revealed that the identified host specificity-related DEGs have diversified roles in symbiosis, meaning that host specific regulation maybe existed in the whole progress of nodule development and senescence, not only in nodulation.

In summary, we firstly combined the expression characteristics of rhizobial genes in symbiosis with the growth and development of soybean, and identified 164 important rhizobial DEGs associated with nodule development and senescence. Among these DEGs, many are firstly identified and showed to associate with nodule development and senescence. Besides, we focused on the DEGs encoding nod, nif, fix proteins and T3SS secretion system-related proteins, as well as proteins involved in nitrogen metabolism, ABC transporters and two-component system pathways. Our results supply valuable basises for studying the genetic determinants of soil rhizobia in symbiosis and the creation of high efficiency soybean cultivation technology.

## Materials and Methods

### Genome and Gene Mapping

The clean reads for the five nodule samples at five developmental stages of soybean (branching stage, flowering stage, fruiting stage, pod stage and harvest stage) were obtained from our previous study (Yuan et al., 2017). The raw sequence reads have been submitted to NCBI under the assigned accession number PRJNA765164, and the BioSample accessions included SAMN21545854, SAMN21545855, SAMN21545856, SAMN21545857, and SAMN21545858. The information of the reference genome (*B. diazoefficiens* 113-2) was previously described (Li et al., 2020). We map clean reads to the reference genome using HISAT (Cock et al., 2010), and map clean reads to reference transcripts using Bowtie2 (Kim et al., 2015). The mapping details are shown in Supplementary Table 1.

### Gene Expression Analysis and Correlation Analysis Between Samples

We calculate the gene expression level for each nodule sample with RSEM (Li and Dewey, 2011). Besides, in order to reflect the gene expression correlation between the nodule samples, we calculated the Pearson correlation coefficients for all gene expression levels between each two samples using *cor* function in R software, and reflected these coefficients in the form of heat maps. Heatmap was performed using TBtools software (Chen et al., 2020).

### Screening of DEGs Between Different Nodule Samples

To screen the DEGs between nodule samples, in combination with the false discovery rata (FDR) ≤ 0.001 and | log2 ratio| ≥ 1, the fold changes in gene expression were used to evaluate the significance of gene expression differences in the ten Groups which are classified according to our previous study (Yuan et al., 2017). Data of the DEGs in the ten Groups between five nodule samples are provided in Supplementary Table 3.

### Gene Ontology Functional and Kyoto Encyclopedia of Genes and Genomes Pathway Analyses of DEGs

Gene Ontology (GO) is a database created by the Gene Ontology Consortium and is a classification system for genes’ biological functions. The method of GO functional enrichment analysis first maps all DEGs to terms in the GO database^2^ and the computing method as previously described (Yuan et al., 2017). Kyoto Encyclopedia of Genes and Genomes (KEGG) is a public pathway-related database,^3^ and the computing method also were described in our previous study (Yuan et al., 2017).

### Orthologs Analysis of the DEGs

Firstly, we performed Core/Pan gene analysis for *B. diazoefficiens* 113-2, *M. loti* MAFF303099, *M. huakuii* 7653R, and *S. meliloti* 2011. We used the CD-HIT 4.66^4^ rapid clustering of similar proteins software (Edgar, 2004)with a threshold of 50% pairwise identity and 0.7 length difference cut off in amino acid to cluster the Core/Pan genes of these four strains, and obtained the pan gene pool. Then we used the identified DEGs as queries to search the orthologs in the pan gene pool.

### Phylogenetic Analysis

Phylogenetic trees were conducted in MEGA X (Kumar et al., 2018). The evolutionary history was inferred using the Neighbor-Joining method (Saitou and Nei, 1987). The percentage of replicate trees in which the associated taxa clustered together in the bootstrap test (1000 replicates) are shown next to the branches.

## Data Availability Statement

The original contributions presented in the study are included in the article/Supplementary Material, further inquiries can be directed to the corresponding author/s.

## Author Contributions

SY, HC, and XZ designed this work. SY wrote the manuscript. SY, SZ, and YF performed most of the analysis, figures, and tables. CZ, YH, ZS, SC, WG, HY, ZY, and DQ contributed substantially to the completion of this work. All authors contributed to the article and approved the submitted version.

## Conflict of Interest

The authors declare that the research was conducted in the absence of any commercial or financial relationships that could be construed as a potential conflict of interest.

## Publisher’s Note

All claims expressed in this article are solely those of the authors and do not necessarily represent those of their affiliated organizations, or those of the publisher, the editors and the reviewers. Any product that may be evaluated in this article, or claim that may be made by its manufacturer, is not guaranteed or endorsed by the publisher.

## Footnotes

1
http://www.kegg.jp/kegg/kegg1.html

2
http://www.geneontology.org/

3
https://www.kegg.jp/kegg/kegg1.html

4
http://weizhong-lab.ucsd.edu/cd-hit/
