# Supplementary material for: Identification of the Important Genes of Bradyrhizobium diazoefficiens 113-2 Involved in Soybean Nodule Development and Senescence
Source: Front Microbiol. 2021 Nov 11;12:754837. doi: 10.3389/fmicb.2021.754837 (PMC8632152; doi:10.3389/fmicb.2021.754837)
Supplement: Supplementary file 9 [file Data_Sheet_1.PDF]

Sequencing saturation (Branching stage_N)

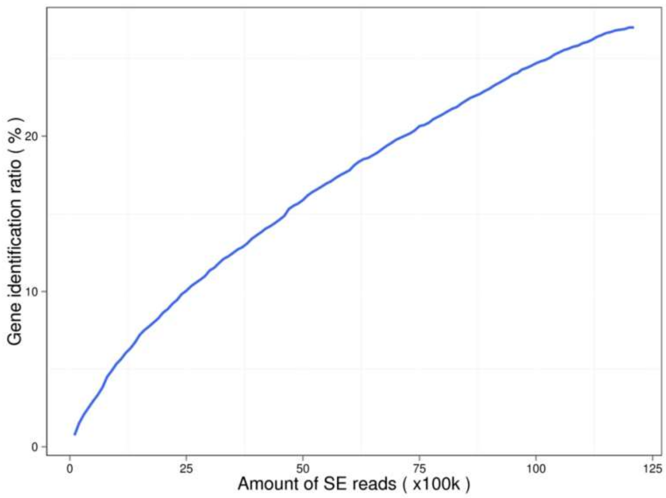

Sequencing saturation (Flowering stage_N)

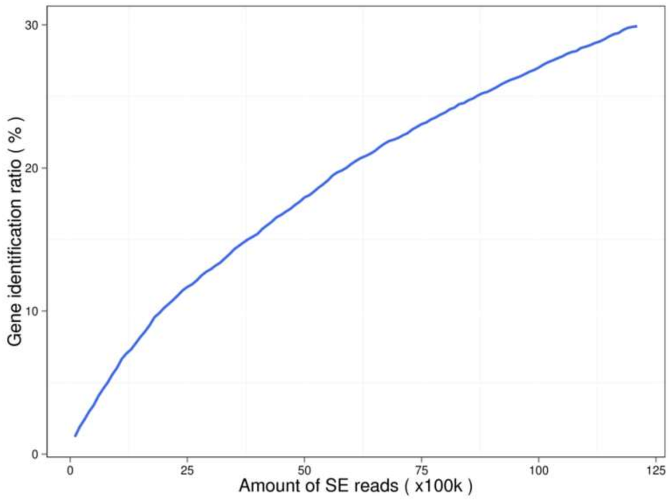

Sequencing saturation (Fruiting stage_N)

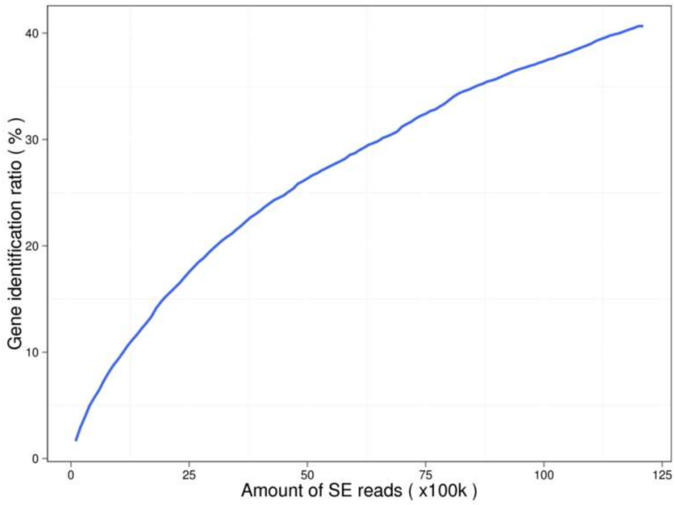

Sequencing saturation (Pod stage_N)

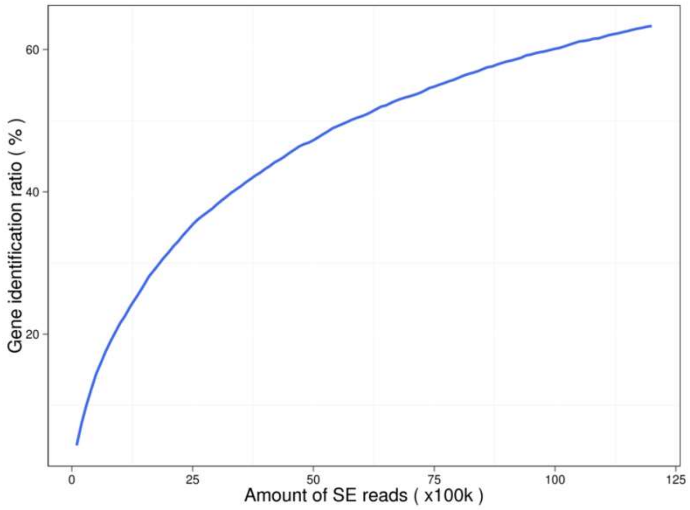

Sequencing saturation (Harvest stage_N)

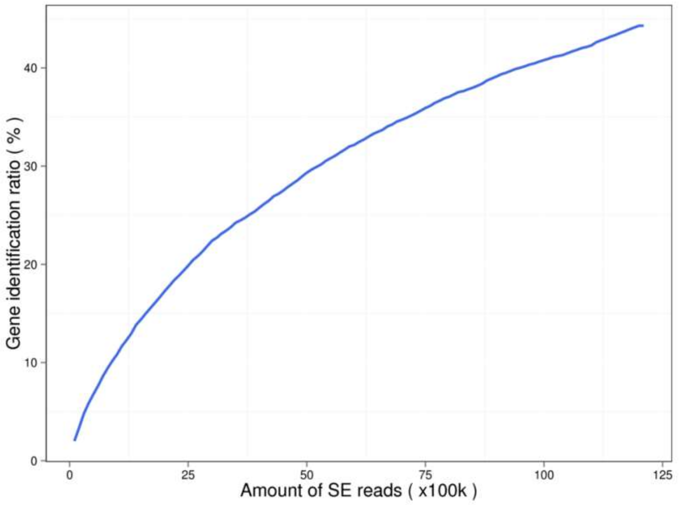

Figure S1: The sequencing saturation analysis of DEGs of *B. diazoefficiens* 113-2.

Reads random (Branching stage_N)

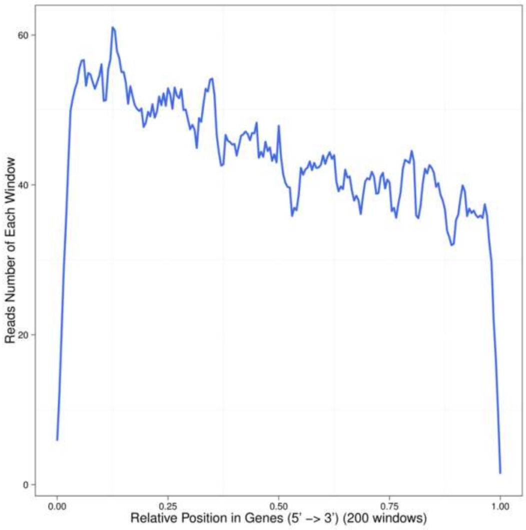

Reads random (Flowering stage_N)

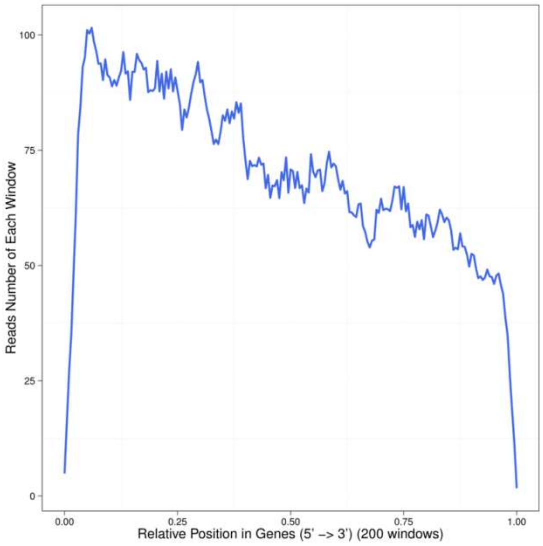

Reads random (Fruiting stage_N)

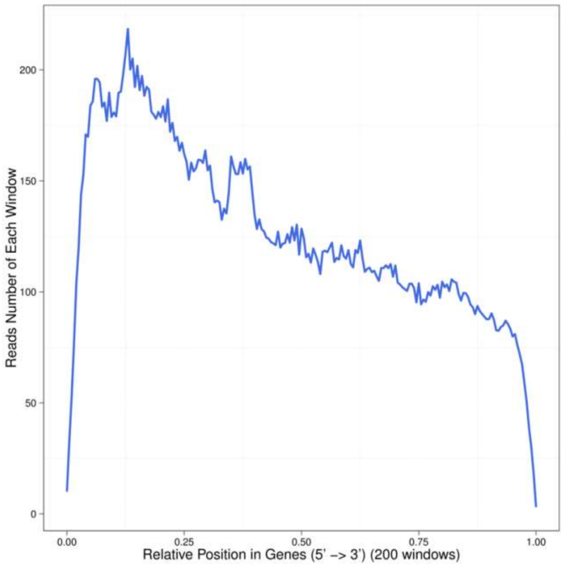

Reads random (Pod stage_N)

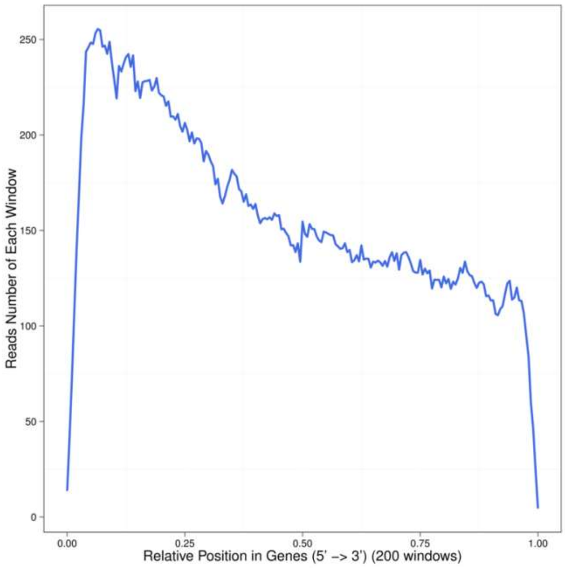

Reads random (Harvest stage_N)

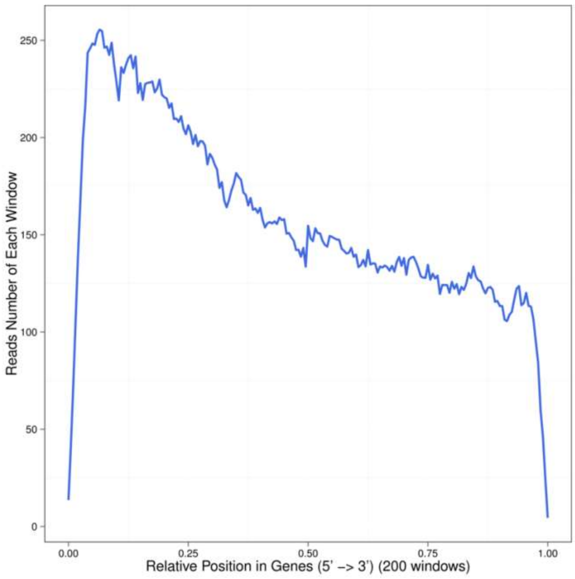

Figure S2: The reads random analysis of DEGs of *B. diazoefficiens* 113-2.

Reads coverage (Branching stage_N)

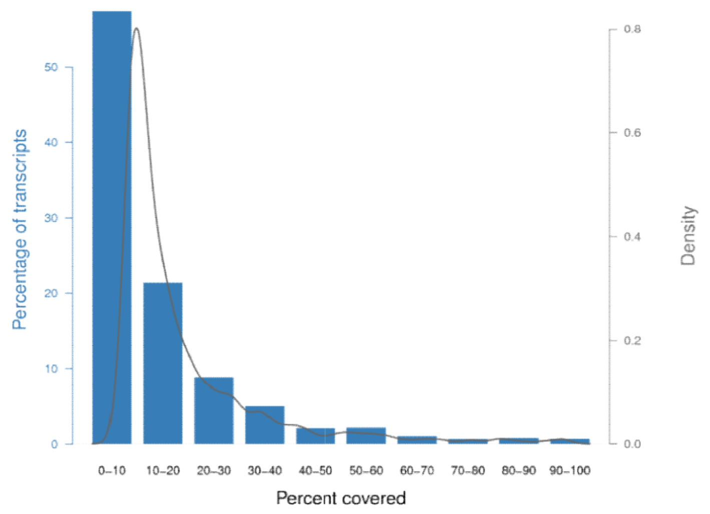

Reads coverage (Flowering stage_N)

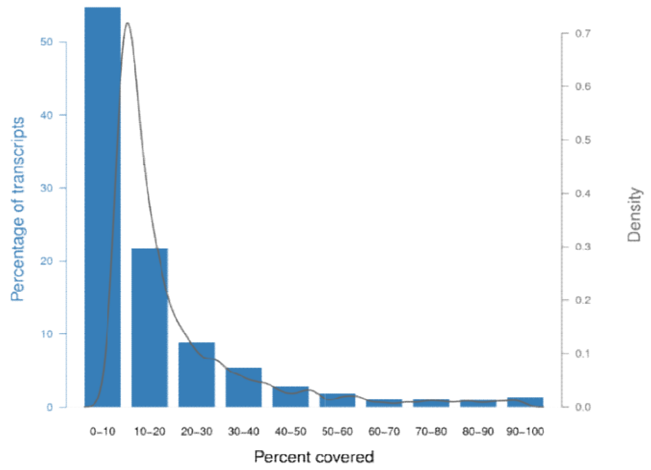

Reads coverage (Fruiting stage_N)

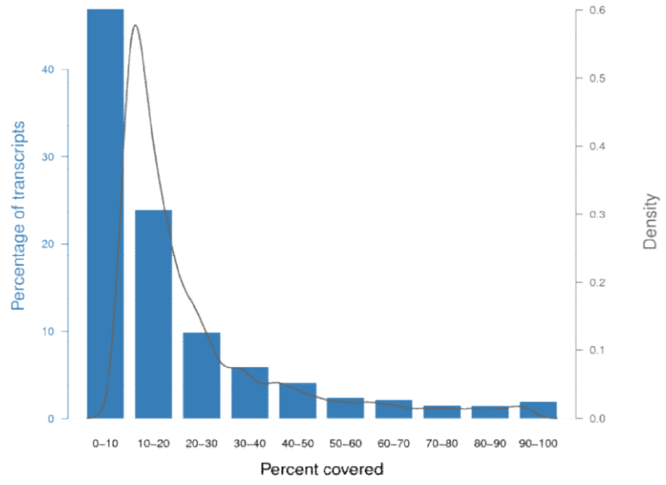

Reads coverage (Pod stage_N)

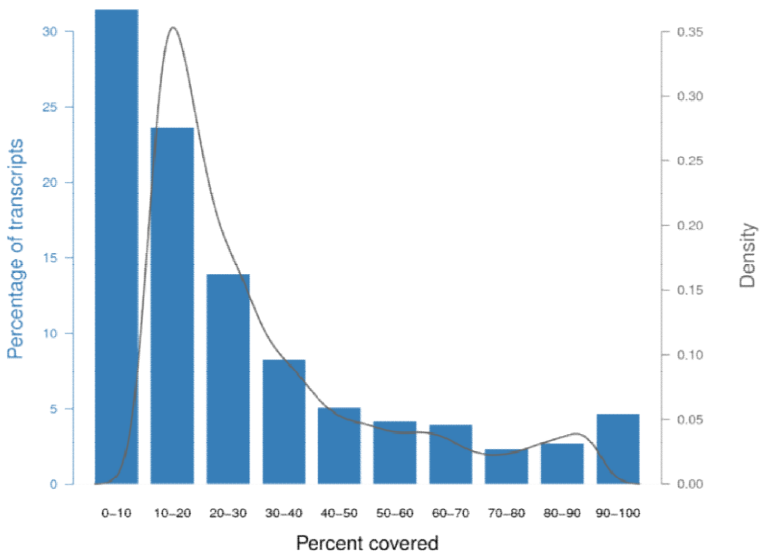

Reads coverage (Harvest stage_N)

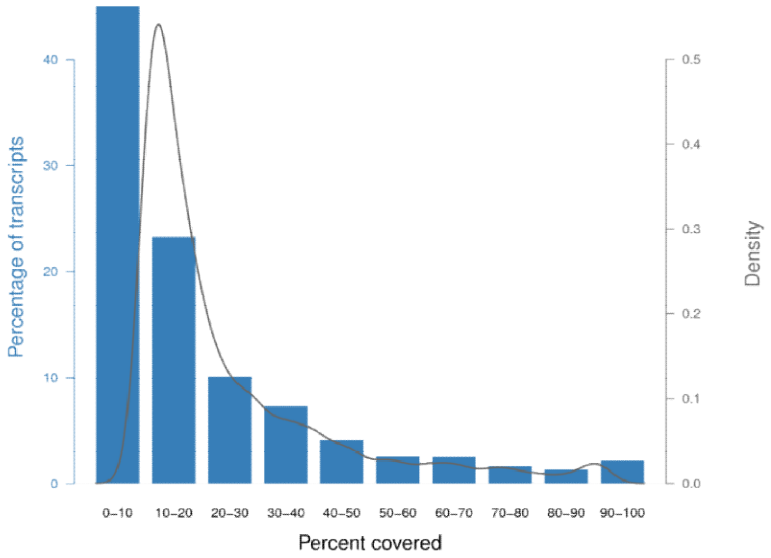

Figure S3: The reads coverage analysis of DEGs of *B. diazoefficiens* 113-2.

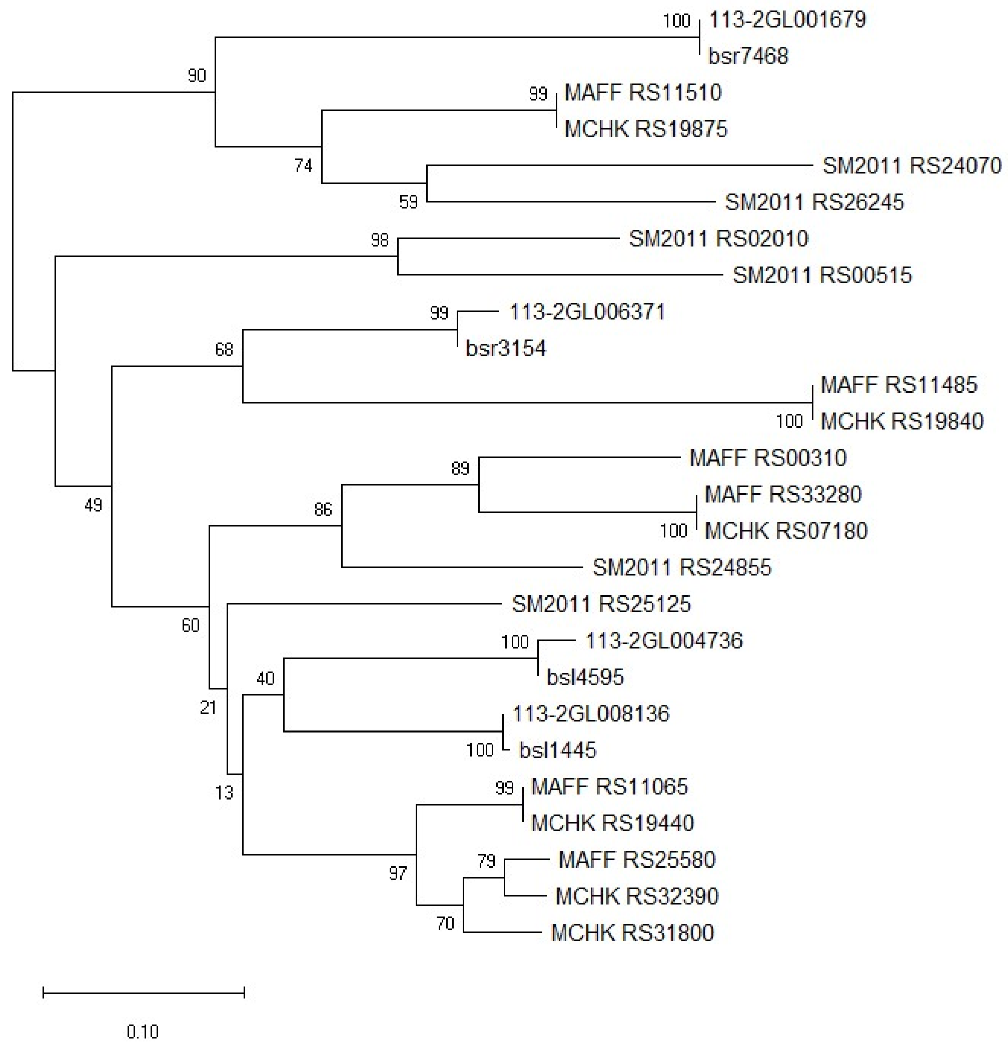

Supplemental Figure S4: Phylogenetic relationships among the orthologs of 113-2GL004736 from the five strains.

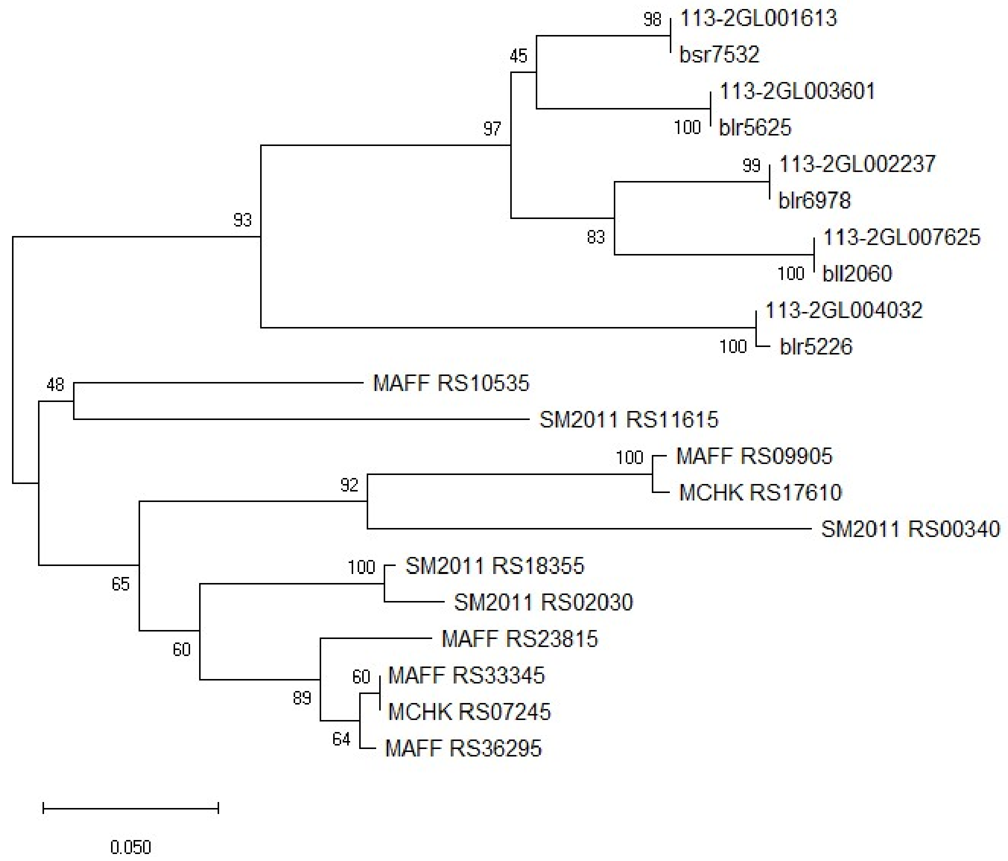

Supplemental Figure S5: Phylogenetic relationships among the orthologs of 113-2GL007625 from the five strains.

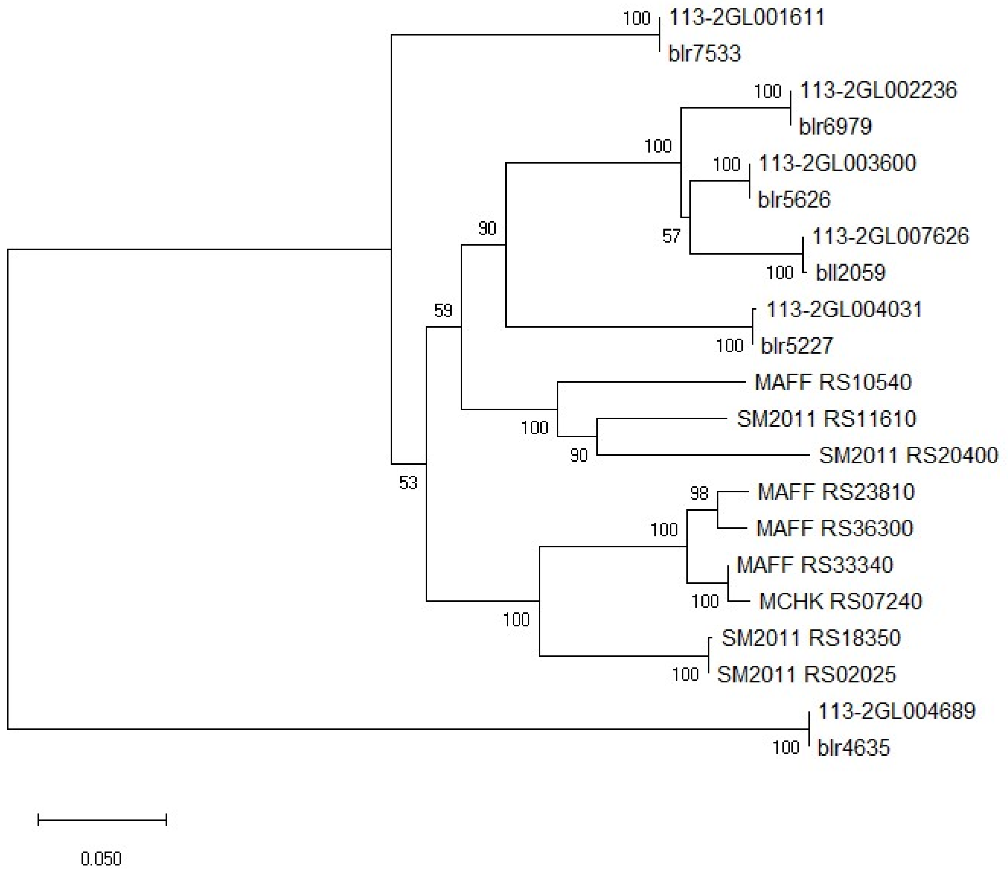

Supplemental Figure S6: Phylogenetic relationships among the orthologs of 113-2GL007626 from the five strains.

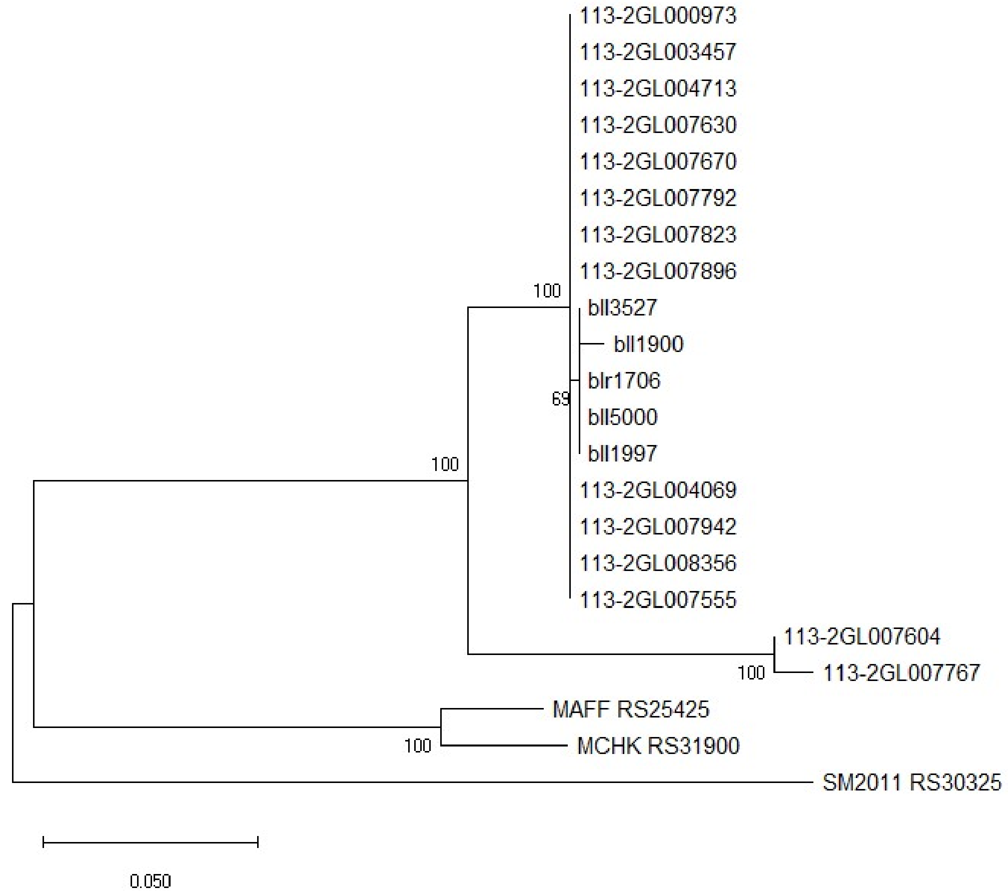

Supplemental Figure S7: Phylogenetic relationships among the orthologs of 113-2GL007604 from the five strains.
